# An Improvised Pulmonary Telerehabilitation Program for Postacute COVID-19 Patients Would Be Feasible and Acceptable in a Low-Resource Setting

**DOI:** 10.1097/PHM.0000000000001666

**Published:** 2021-01-04

**Authors:** Fanuel Meckson Bickton, Enock Chisati, Jamie Rylance, Ben Morton

**Affiliations:** From the University College London, London, United Kingdom (FMB); Malawi-Liverpool-Wellcome Trust Clinical Research Programme, Blantyre, Malawi (FMB, JR, BM); College of Medicine, University of Malawi, Physiotherapy Department, Blantyre, Malawi (EC); Liverpool School of Tropical Medicine, Liverpool, United Kingdom (JR, BM); and Aintree University Hospital NHS Foundation Trust, Liverpool, United Kingdom (BM).

**Keywords:** COVID-19, Physiotherapy, Pulmonary Rehabilitation, Telerehabilitation, Low-Resource Setting

## Abstract

Postacute COVID-19 patients are at risk of long-term functional impairment, and the rehabilitation community is calling for action preparing for a “tsunami of rehabilitation needs” in this patient population. In the absence of standard guidelines and local evidence, a 3-wk pulmonary telerehabilitation program was successfully delivered to a postacute severe COVID-19 patient in Malawi. The patient experienced persistent dyspnea and fatigue, with a remarkable impact on his health status. On the final assessment, all his respiratory severity scores had fallen by more than their thresholds for clinical significance. He reported no continued or new complaints, was walking longer distances, had returned to work, and was discharged from follow-up. Our case shows that an improvised pulmonary telerehabilitation program for postacute COVID-19 patients could be feasible and acceptable in a low-resource setting. Benefits include reducing risk of transmission and use of personal protective equipment.

## Abstract 2

Supplemental digital content is available in the text.

## BACKGROUND

As COVID-19 patients are discharged successfully from the acute hospital setting, a “tsunami of rehabilitation needs” is anticipated.^1^ Our aim was to define a rehabilitation program for postacute COVID-19 patients in a low-resource setting, based on an existing algorithm of pulmonary rehabilitation for chronic respiratory disease.^2^ However, physical distancing requirements to prevent COVID-19 transmission challenge the delivery of traditional face-to-face pulmonary rehabilitation paradigms and prompted us to explore a telehealth intervention.^3^

Telerehabilitation uses information and communication technologies to deliver clinical rehabilitation services from distance.^4^ It can reduce direct contact between rehabilitation professionals and patients, reducing COVID-19 transmission risk and use of personal protective equipment.^5^ In countries affected early by the pandemic, such as Italy, the need for specific rehabilitation is apparent because of impaired physical function and impaired performance of activities of daily living.^6^

As of November 10, 2020, confirmed cases in Malawi had cumulatively reached 5953, with 185 deaths.^7^ With the current number of recovered cases reportedly overtaking the number of active cases, the country will potentially move into a different phase of the pandemic where the number of postacute COVID-19 patients requiring rehabilitation services will increase. Presented here is a case of a successful improvised pulmonary telerehabilitation program in a patient recovering from severe COVID-19 in this low-resource setting. Currently, there is limited pulmonary rehabilitation capability in Malawi owing to a shortage of qualified healthcare professionals and expertise. Therefore, our patient is one of the few patients to have benefited from such an intervention.

This case report conforms to all CAse REports guidelines^8^ and reports the required information accordingly (see Supplemental Checklist, Supplemental Digital Content 1, http://links.lww.com/PHM/B191). Appropriate written informed consent was obtained for the publication of this case report.

## CASE PRESENTATION

On June 29, 2020, a premorbidly well 46-yr-old man was successfully discharged after 10 days of hospital admission due to severe COVID-19 infection. Information about the patient’s clinical history, examination, course, and management during his hospitalization was previously reported on elsewhere^9^ by another group of authors, in the acute hospital setting. In line with World Health Organization guidelines,^10^ he was self-isolating at home during early convalescence after discharge.

To identify the patient’s rehabilitation needs, an initial assessment was performed via a WhatsApp video call on the fifth day of the patient’s home confinement. Assessed were the patient’s perceived respiratory disability due to dyspnea (using the modified Medical Research Council [mMRC] dyspnea scale^11^), health status impairment (using the Chronic Obstructive Pulmonary Disease Assessment Test [CAT]^12^), and subjective experience of fatigue (using the Checklist Individual Strength fatigue subscale [CIS-Fatigue]^13^). At baseline, the patient scored 3 on the mMRC dyspnea scale and 8 on CAT. According to the Global Initiative for Chronic Obstructive Lung Disease guidelines,^14^ an mMRC score of 2 or higher or a CAT score of 10 or higher is indicative that dyspnea was a significant symptom, with a remarkable impact on health status. His CIS-Fatigue score of 43 exceeded the threshold for “severe fatigue” (>35).^15^

**FIGURE 1:**
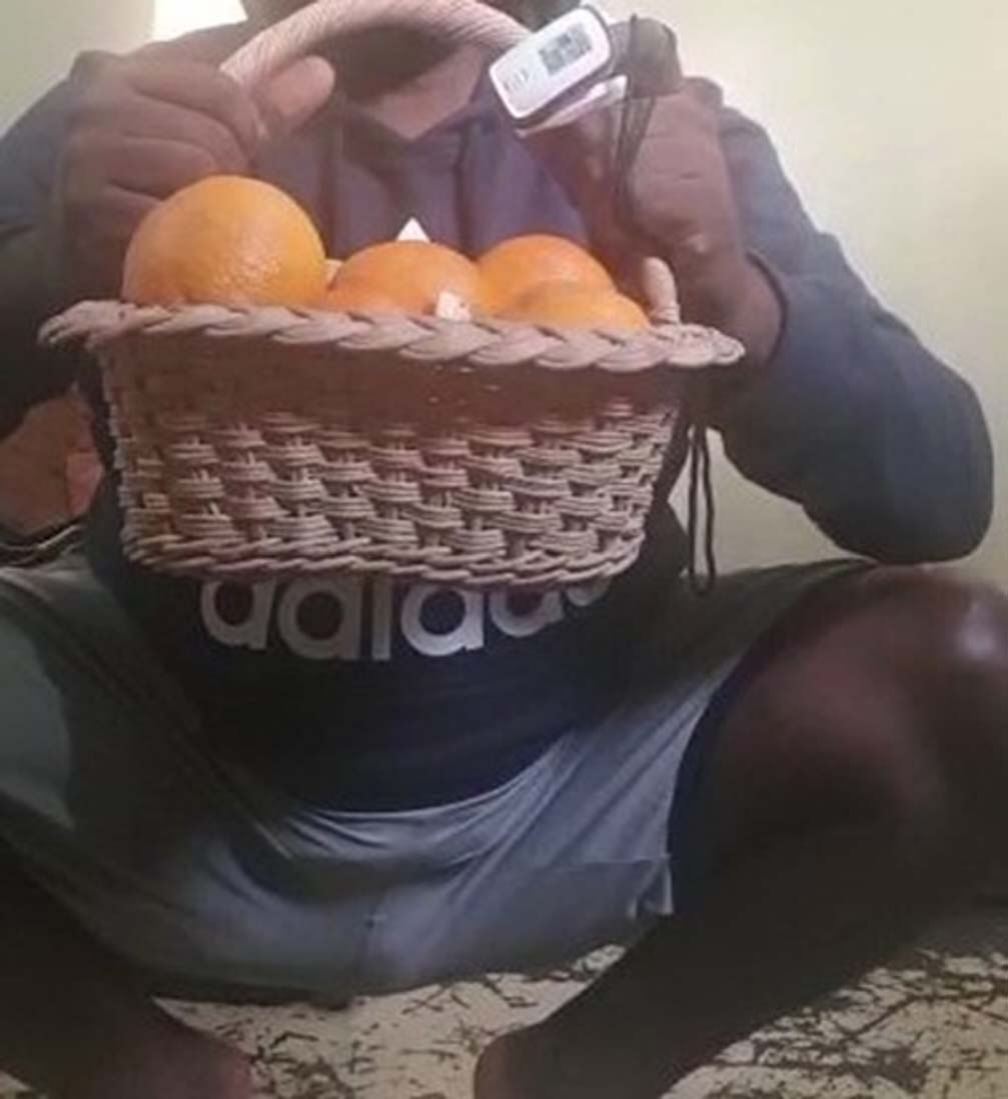
Patient performing a goblet squat with weighted overhead reach, improvised with a basket filled with objects including fruits.

Subsequently, a pulmonary telerehabilitation program was designed, implemented, and supervised by the first author, who is a qualified physiotherapist registered with the Medical Council of Malawi and certified in pulmonary rehabilitation jointly by the American Association of Cardiovascular and Pulmonary Rehabilitation and the American Association for Respiratory Care. Delivery and supervision were conducted via WhatsApp text messaging, video, and audio calls. The program ran over 3 wks and consisted of education and patient-tailored progressive exercise sessions. Education sessions included topics on the COVID-19 disease process and importance of exercising. Exercise sessions included breathing, aerobic, and strength training. Breathing training consisted of pursed lip breathing (initially for 5 mins with 3-sec breath hold) performed either independently or during rest periods between other exercises. Aerobic training included marching on the spot building to low-level knee raises and walking around a room (initially for 5 mins). Each session lasted for 15 to 30 mins. To build up the results, the program was progressed over time as guided by the patient’s perceived rate of exertion. This included increasing the duration of the exercises (e.g., from 5 to 8 mins of marching on the spot and pursed lip breathing), raising knees to the waist level and increasing speed during marching, and adding a strength training exercise—goblet squat with weighted overhead reach (Fig. 1).

**FIGURE 2:**
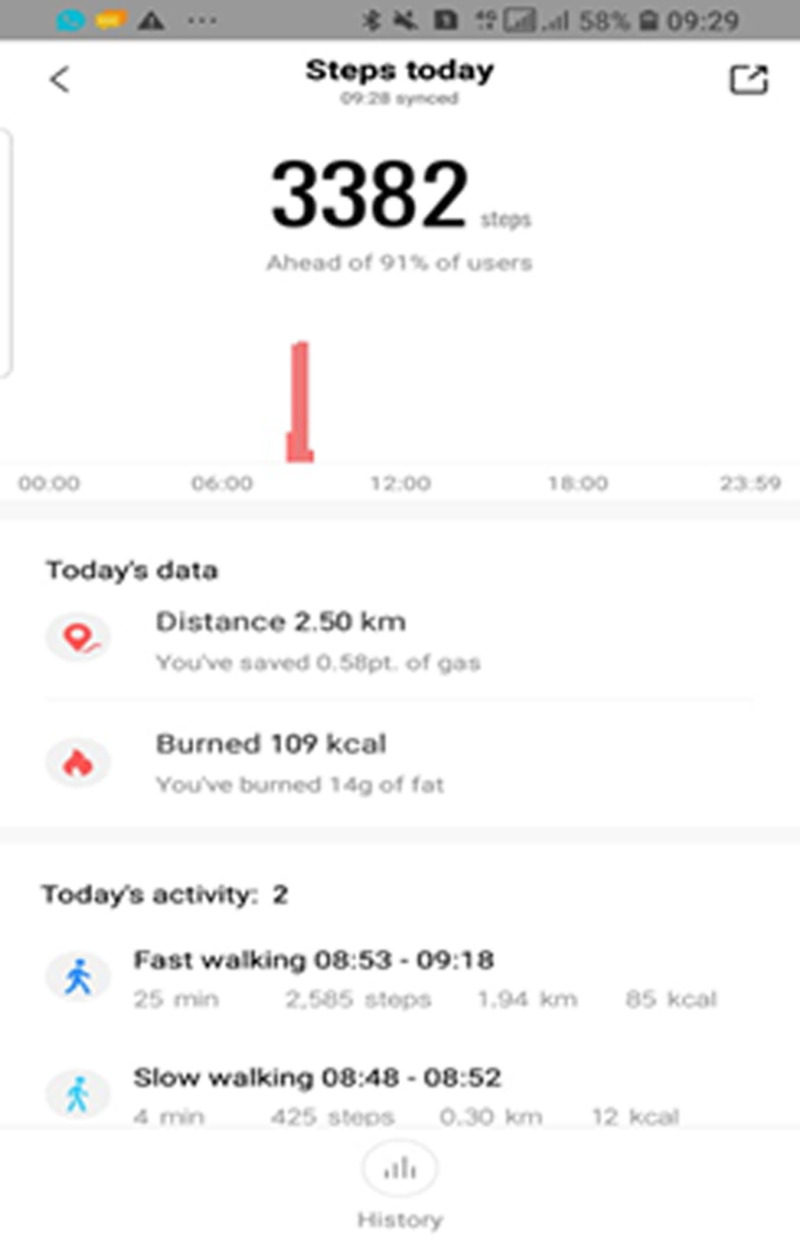
A screenshot of the pedometer recordings taken by the patient during one of his outdoor walking sessions (within the initial 6 days postisolation).

The patient was coached to perform exercises for at least 2 sessions per day and 3 days per week. Exercise progression was tailored for the patient over continued assessments. To observe the social distancing and self-isolation mandate, the patient initially performed the exercises inside his confinement room. On the first day after self-isolation, the exercise regimen was progressed to outdoor walking up to 300 m. The patient used the pursed lip breathing as a self-management strategy during episodes of dyspnea. Later, outdoor walking was progressed to more than 2500 m per day and the patient returned to work during the third week of the program. Within the initial 6 days of postisolation period, the patient achieved 2577 m for 29 mins consisting of 25 mins of fast walking for 1970 m and 4 mins of slow walking for 324 m—all figures rounded to the nearest integer (Fig. 2).

In total, the rehabilitation program ran for 3 wks (from day 1 of the program during the isolation period to the discharge day of the program in the postisolation period), achieving a total of 9 or more sessions. During the final assessment (on discharge day), all respiratory severity scores had fallen by more than their thresholds for clinical significance (Table 1). At this point, the patient reported no continued or new complaints, also adding in his own words, “They [the exercises] have helped 100%.” He was now walking longer distances, had returned to work, and was discharged from follow-up with encouragement to maintain his physically active lifestyle.

**TABLE 1:** Assessment outcomes before and after rehabilitation

Outcomes	Outcome Measure	Outcome Scores		Clinically Important Difference?
		Before Rehabilitation	After Rehabilitation	
Dyspnea	mMRC	3	1	Yes
Health status impairment	CAT	8	2	Yes
Fatigue	CIS-Fatigue	43	11	Yes

mMRC measures functional limitation resulting from dyspnea; CAT measures globally the impact of cough, sputum, dyspnea, and chest tightness on health status/health-related quality of life; and CIS-Fatigue measures four dimensions of fatigue: fatigue severity, concentration problems, reduced motivation and activity. The unit change indicative of a clinically important difference is 1 for mMRC,^16^ 2 for CAT,^17^ and 9 for CIS-Fatigue.^18^

## DISCUSSION

Survivors of severe COVID-19 are at risk of developing long-term functional impairment, with exertional dyspnea widely reported.^19^ We acknowledge a limited evidence base for pulmonary rehabilitation in post-acute COVID-19 patients, and home-based pulmonary telerehabilitation in this patient population is a field that is yet to be systematically implemented. Therefore, we lacked standard guidelines and local evidence; these challenges are also reported elsewhere.^20^

The current COVID-19 Interim Guidance on Rehabilitation in the Hospital and Post-Hospital Phase^21^ acknowledges that the existing data from survivors of viral pneumonias indicate a wide range of challenges that patients face and it is unlikely that a unidimensional program of rehabilitation will meet the needs of the COVID-19 survivor as they will exhibit multiple treatable traits that a comprehensive rehabilitation program has the potential to modify favorably. However, we also agree with the authors of this guidance that, although data on safety and efficacy are lacking, we (healthcare professionals) cannot wait for published research evidence before we can start these rehabilitative interventions in our daily clinical practice, owing to the rapidly increasing number of post-COVID-19 patients.

We formulated an approach to mirror an algorithm of pulmonary rehabilitation developed for patients with well-known chronic respiratory conditions, especially chronic obstructive pulmonary disease.^2^ The reported overlap in many symptoms between postacute COVID-19 patients and the more traditional candidates for pulmonary rehabilitation (including those with chronic obstructive pulmonary disease) is acknowledged,^22^ and the model of pulmonary rehabilitation currently suits as a framework, particularly in a subset of post-acute COVID-19 patients with persistent symptoms like our patient.^21^ However, in our resource-limited setting (limited equipment and technical skills), we were unable to deliver a formal comprehensive interdisciplinary pulmonary telerehabilitation program, as done in high-income countries.^23,24^ For example, to deliver telerehabilitation, we used WhatsApp on personal mobile phones rather than more advanced hardware and software available to teams in higher-income countries.^23^ Because of lack of equipment (except for later exercise sessions when the patient managed to access and use a finger pulse oximeter), we were also unable to objectively monitor the patient’s physiologic responses to exercise, such as heart rate and oxygen saturation; this could potentially compromise patient safety during exertional exercises. We therefore focused our assessment on patient-reported subjective exercise tolerance during assessments, including perceived rate of exertion, symptoms of dizziness, and intolerable shortness of breath, and took a conservative approach to exercise progression. Although this approach precluded assessment metrics such as the incremental shuttle walk test, we were able to use a combination of mMRC, CAT, and CIS-Fatigue scores to longitudinally measure response to the exercise program. We recommend that this approach is pragmatic and deliverable in low-income settings where smartphone ownership is increasing,^25^ and paired before and after measurements should be considered to objectively measure the efficacy of physiotherapy interventions.

## CONCLUSION

We propose that telerehabilitation is a viable alternative to traditional face-to-face intervention. Our case shows that an improvised pulmonary telerehabilitation program for postacute COVID-19 patients could be feasible and acceptable in a low-resource setting. These initial observations require corroboration by more high-quality studies. Notwithstanding, we recommend telerehabilitation services to be part of Malawi’s national response to COVID-19. Besides helping to reduce the risk of transmission and use of personal protective equipment, telerehabilitation would make efficient use of the nation’s critically limited pool of rehabilitation professionals. Patients and their caregivers would also incur fewer costs in travel to a healthcare facility to access services. Above all, it reminds us that innovation can be driven by adversity, and that the determination of healthcare workers in low-income countries, as elsewhere, can significantly improve patients’ lives.

## Supplementary Material

SUPPLEMENTARY MATERIAL
