# Supplementary material for: An Improvised Pulmonary Telerehabilitation Program for Postacute COVID-19 Patients Would Be Feasible and Acceptable in a Low-Resource Setting
Source: Am J Phys Med Rehabil. 2021 Jan 4;100(3):209–12. doi: 10.1097/PHM.0000000000001666 (PMC7886367; doi:10.1097/PHM.0000000000001666)
Supplement: SUPPLEMENTARY MATERIAL [file ajpmr-100-209-s001.docx]

**An improvised pulmonary tele-rehabilitation programme for post-acute COVID-19 patients would be feasible and acceptable in a low-resource setting: a case report**

Fanuel Meckson Bickton^1,2^, Enock Chisati^3^, Jamie Rylance^2,4^, Ben Morton^2,4,5^

1. University College London, Gower Street, London, WC1E 6BT, United Kingdom
2. Malawi-Liverpool-Welcome Trust Clinical Research Programme, Blantyre, Malawi
3. University of Malawi, College of Medicine, Physiotherapy Department, Blantyre, Malawi
4. Liverpool School of Tropical Medicine, Pembroke Place, Liverpool, L3 5QA, United Kingdom
5. Aintree University Hospital NHS Foundation Trust, Lower Lane, Fazakerley, Liverpool, L9 7AL, United Kingdom

**Correspondence to:**

Fanuel Meckson Bickton, University College London, Gower Street, London, WC1E 6BT, United Kingdom ([fanuel.bickton.20@ucl.ac.uk](mailto:fanuel.bickton.20@ucl.ac.uk); [fbickton@mlw.mw](mailto:fbickton@mlw.mw); [fmbickton@stud.medcol.mw](mailto:fmbickton@stud.medcol.mw))

Main text word count:1,449

Abstract word count: 143

Number of tables: 1

Number of figures: 2

Number of references: 25

**Funding:** This work was supported by the UK Foreign, Commonwealth and Development Office and Wellcome [220757/Z/20/Z].

**Abstract:** Post-acute COVID-19 patients are at risk of long-term functional impairment and the rehabilitation community is calling for action preparing for a “tsunami of rehabilitation needs” in this patient population. In the absence of standard guidelines and local evidence, we successfully delivered a three-week pulmonary tele-rehabilitation programme to a post-acute severe COVID-19 patient in Malawi. The patient suffered from persistent dyspnoea and fatigue, with a remarkable impact on his health status. On the final assessment, all his respiratory severity scores had fallen by more than their thresholds for clinical significance. He reported no continued or new complaints, was walking longer distances, had returned to work, and was discharged from follow-up. Our case shows that an improvised pulmonary tele-rehabilitation programme for post-acute COVID-19 patients could be feasible and acceptable in a low-resource setting. Benefits include reducing risk of transmission and use of personal protective equipment.

**Key Words:** COVID-19, physiotherapy, pulmonary rehabilitation, tele-rehabilitation, low-resource setting

**BACKGROUND**

As COVID-19 patients are discharged successfully from the acute hospital setting, a “tsunami of rehabilitation needs” is anticipated^1^. Our aim was to define a rehabilitation programme for post-acute COVID-19 patients in a low-resource setting, based on an existing algorithm of pulmonary rehabilitation for chronic respiratory disease.^2^ However, physical distancing requirements to prevent COVID-19 transmission challenge the delivery of traditional face-to-face pulmonary rehabilitation paradigms and prompted us to explore a telehealth intervention.^3^

Tele-rehabilitation employs information and communication technologies to deliver clinical rehabilitation services from distance.^4^ It can reduce direct contact between rehabilitation professionals and patients, reducing COVID-19 transmission risk and use of personal protective equipment (PPE).^5^ In countries affected early by the pandemic, such as Italy, the need for specific rehabilitation is apparent due to impaired physical function and impaired performance of activities of daily living.^6^

As of 10 November 2020, confirmed cases in Malawi had cumulatively reached 5,953with 185 deaths.^7^ With the current number of recovered cases reportedly overtaking the number of active cases, the country will potentially move into a different phase of the pandemic where the number of post-acute COVID-19 patients requiring rehabilitation services will increase. We present a case of a successful improvised pulmonary tele-rehabilitation programme in a patient recovering from severe COVID-19 in this low-resource setting. Currently, there is a limited pulmonary rehabilitation capability in Malawi due to shortage of qualified health care professionals and expertise. Therefore, our patient is one of the few patients to have benefited from such an intervention.

This case report conforms to all CARE guidelines^8^ and reports the required information accordingly (see Supplemental Checklist, Supplemental Digital Content 1, http://links.lww.com/PHM/B191). Appropriate written informed consent was obtained for publication of this case report.

**CASE PRESENTATION**

On 29 June 2020, a pre-morbidly well 46-year old male was successfully discharged after 10 days of hospital admission due to severe COVID-19 infection. Information about the patient’s clinical history, examination, course, and management during his hospitalisation was previously reported on elsewhere^9^ by another group of authors, in the acute hospital setting. In line with WHO guidelines,^10^ he was self-isolating at home during early convalescence after discharge.

To identify the patient’s rehabilitation needs, we performed an initial assessment via a WhatsApp video call on the fifth day of the patient’s home confinement. We assessed the patient’s perceived respiratory disability due to dyspnoea (using the modified Medical Research Council (mMRC) dyspnoea scale^11^), health status impairment (using the Chronic Obstructive Pulmonary Disease (COPD) Assessment Test (CAT)^12^), and subjective experience of fatigue (using the Checklist Individual Strength (CIS) fatigue subscale^13^). At baseline, the patient scored three on mMRC dyspnoea scale, and eight on CAT. According to the Global Initiative for Chronic Obstructive Lung Disease (GOLD) guidelines^14^, an mMRC score of ≥2 or a CAT score of ≥10 is indicative that dyspnoea was a significant symptom, with a remarkable impact on health status. His CIS-Fatigue score of 43 exceed the threshold for “severe fatigue” (>35).^15^

Subsequently, a pulmonary tele-rehabilitation programme was designed, implemented, and supervised by the first author who is a qualified physiotherapist registered with the Medical Council of Malawi and certified in pulmonary rehabilitation jointly by the American Association of Cardiovascular and Pulmonary Rehabilitation (AACVPR) and the American Association for Respiratory Care (AARC). Delivery and supervision were conducted via WhatsApp text-messaging, video, and audio calls. The programme ran over three weeks and consisted of education and patient-tailored progressive exercise sessions. Education sessions included topics on COVID-19 disease process and importance of exercising. Exercise sessions included breathing, aerobic, and strength training. Breathing training consisted of pursed lip breathing (initially for five minutes with three seconds breath hold) performed either independently or during rest periods between other exercises. Aerobic training included marching on the spot building to low-level knee raises and walking around a room (initially for five minutes). Each session lasted for 15 to 30 minutes. To build up the results, the programme was progressed over time as guided by the patient’s perceived rate of exertion (PRE). This included increasing the duration of the exercises (e.g., from five to eight minutes of matching on the spot and pursed lip breathing), raising knees to the waist level and increasing speed during matching, and addition of a strength training exercise – goblet squat with weighted overhead reach (Figure 1).

(Figure 1. Patient performing a goblet squat with weighted overhead reach, improvised with a basket filled with objects including fruits)

The patient was coached to perform exercises for at least two sessions per day and three days per week. Exercise progression was tailored for the patient over continued assessments. To observe the social distancing and self-isolation mandate, the patient initially performed the exercises inside his confinement room. On the first day post-self-isolation, the exercise regimen was progressed to outdoor walking up to 300 metres. The patient used the pursed lip breathing as a self-management strategy during episodes of dyspnoea. Later, outdoor walking was progressed to over 2500 meters per day and the patient returned to work during the third week of the programme. Within the initial six days of post-isolation period, the patient achieved 2577 meters for 29 minutes consisting of 25 minutes of fast walking for 1970 meters and 4 minutes of slow walking for 324 meters – all figures rounded to the nearest integer (Figure 2).

(Figure 2. A screenshot of the pedometer recordings taken by the patient during one of his outdoor walking sessions (within the initial six days post-isolation)

In total, the rehabilitation programme run for three weeks (from day one of the programme during the isolation period to the discharge day of the programme in the post-isolation period), achieving a total of ≥9 sessions. During the final assessment (on discharge day), all respiratory severity scores had fallen by more than their thresholds for clinical significance (see Table 1). At this point, the patient reported no continued or new complaints, also adding in his own words, “They [the exercises] have helped 100%.” He was now walking longer distances, had returned to work, and was discharged from follow-up with encouragement to maintain his physically active lifestyle.

(Table 1. Assessment outcomes before and after rehabilitation)

**DISCUSSION**

Survivors of severe COVID-19 are at risk of developing long-term functional impairment with exertional dyspnoea widely reported.^19^ We acknowledge a limited evidence base for pulmonary rehabilitation in post-acute COVID-19 patients and implementation of home-based pulmonary tele-rehabilitation in this patient population is a field that is yet to be systematically implemented. Therefore, we lacked standard guidelines and local evidence; these challenges are also reported elsewhere.^20^

The current COVID-19 Interim Guidance on Rehabilitation in the Hospital and Post-Hospital Phase^21^ acknowledge that the existing data from survivors of viral pneumonias indicate a wide range of challenges that patients face and it is unlikely that a unidimensional programme of rehabilitation will meet the needs of the Covid-19 survivor as they will exhibit multiple treatable traits that a comprehensive rehabilitation programme has the potential to modify favourably. However, we also agree with the authors of this guidance that, while data on safety and efficacy are lacking, we (healthcare professionals) cannot wait for published research evidence before we can start these rehabilitative interventions in our daily clinical practice, due to the rapidly increasing number of post-COVID-19 patients.

We formulated an approach to mirror an algorithm of pulmonary rehabilitation developed for patients with well-known chronic respiratory conditions, especially chronic obstructive pulmonary disease (COPD).^2^ The reported overlap in many symptoms between post-acute COVID-19 patients and the more traditional candidates for pulmonary rehabilitation (including those with COPD) is acknowledged^22^, and the model of pulmonary rehabilitation currently suits as a framework, particularly in a subset of post-acute COVID-19 patients with persistent symptoms like our patient.^21^ However, in our resource-limited setting (limited equipment and technical skills), we were unable to deliver a formal comprehensive interdisciplinary pulmonary tele-rehabilitation program, as done in high income countries.^23,24^ For example, to deliver tele-rehabilitation, we used WhatsApp on personal mobile phones rather than more advanced hardware and software available to teams in higher-income countries.^23^ Due to lack of equipment (except for later exercise sessions when the patient managed to access and use a finger pulse oximeter), we were also unable to objectively monitor the patient’s physiological responses to exercise such as heart rate and oxygen saturation; this could potentially compromise patient safety during exertional exercises. We had, therefore, focused our assessment on patient-reported subjective exercise tolerance during assessments, including perceived rate of exertion (PRE), symptoms of dizziness and intolerable shortness of breath, and took a conservative approach to exercise progression. Whilst this approach precluded assessment metrics such as the incremental shuttle walk test (ISWT), we were able to employ a combination of mMRC, CAT and CIS-Fatigue scores to longitudinally measure response to the exercise programme. We recommend that this approach is pragmatic and deliverable in low-income settings where smartphone ownership is increasing,^25^ and paired before and after measurements should be considered to objectively measure the efficacy of physiotherapy interventions.

**CONCLUSION**

We propose that tele-rehabilitation is a viable alternative to traditional face-to-face intervention. Our case shows that an improvised pulmonary tele-rehabilitation programme for post-acute COVID-19 patients could be feasible and acceptable in a low-resource setting. These initial observations require corroboration by more high-quality studies. Notwithstanding, we recommend tele-rehabilitation services to be part of Malawi’s national response to COVID-19. Besides helping to reduce the risk of transmission and use of PPE, tele-rehabilitation would make efficient use of the nation’s critically limited pool of rehabilitation professionals. Patients and their caregivers would also incur fewer costs in travel to a healthcare facility to access services. Above all, it reminds us that innovation can be driven by adversity, and that the determination of healthcare workers in low-income countries, as elsewhere, can significantly improve patients’ lives.

**Acknowledgements** The authors thank the patient concerned for providing consent for publication of this report, and Dr. Yohane Gadama for peer-reviewing the report before journal submission.

**Twitter** Fanuel Meckson Bickton @fmbickton, Ben Morton @benjamesmorton, and Jamie Rylance @JamieRylance

**Contributors** FMB led the patient management with supervision from EC, JR, and BM. FMB planned the article and wrote the first draft. EC, JR, and BM critically appraised and edited the draft. All authors approved the final draft.

**Competing interests** None declared.

**Patient consent for publication** Obtained.

**ORCID iDs**

Fanuel Meckson Bickton <https://orcid.org/0000-0002-0925-909X>

Ben Morton <https://orcid.org/0000-0002-6164-2854>

Jamie Rylance <https://orcid.org/0000-0002-2323-3611>

**REFERENCES**

1. Thornton J. Covid-19: the challenge of patient rehabilitation after intensive care. BMJ. 2020;369:m1787. DOI: 10.1136/bmj.m1787
2. Polastri M, Nava S, Clini E, Vitacca M, Gosselink R. COVID-19 and pulmonary rehabilitation: preparing for phase three. Eur Respir J 2020; 55: 2001822. <https://doi.org/10.1183/13993003.01822-2020>
3. Vidal-AlaballaJ, Acosta-Roja R, Hernándeze NP, Luquee US, Morrisone D, Pérez SN. Telemedicine in the face of the COVID-19 pandemic. Aten Primaria. 2020;52(6):418-422. DOI: 10.1016/j.aprim.2020.04.003
4. World Health Organization. Global diffusion of eHealth: making universal health coverage achievable. Report of the third global survey on eHealth2016. Licence: CC BY‐NC‐SA 3.0 IGO
5. Lee A, Goldstein R. The role of telemedicine. Controversies in COPD. European Respiratory Society, 2015. DOI: 10.1183/2312508X.erm6915
6. Belli S, Balbi B, Prince I, Cattaneo D, Masocco F, Zaccaria S, et al. Low physical functioning, and impaired performance of activities of daily life in COVID-19 patients who survived the hospitalisation. Eur Respir J 2020; in press (<https://doi.org/10.1183/13993003.02096-2020>)
7. Republic of Malawi – Ministry of Health. COVID-19 Situation update as of 17th August 2020 [Internet]. 2020, Aug 12 [cited 2020, Aug 12]. Available from: <https://www.facebook.com/malawimoh/>
8. Gagnier JJ, Kienle G, Altman DG, Moher D, Sox H, Riley D; the CARE Group. The CARE guidelines: consensus-based clinical case reporting guideline development. Dtsch Arztebl Int. 2013; 110(37):603-8. DOI: 10.3238/arztebl.2013.0603
9. Banda N, Hara W, Cocker D, Musasa S, Burke RM, Brown C, et al. First case report of a successfully managed severe COVID-19 infection in Malawi. Malawi Med J. 2020 (in press, available at <https://www.mmj.mw/first-case-report-of-a-successfully-managed-severe-covid-19-infection-in-malawi/>)
10. WHO. Clinical management of COVID-19 interim guidance. <https://www.who.int/publications/i/item/clinical-management-of-covid-19>. Published 2020. Accessed 29/07/2020.
11. Bestall JC, Paul EA, Garrod R, Garnham R, Jones PW, Wedzicha JA. Usefulness of the Medical Research Council (MRC) dyspnoea scale as a measure of disability in patients with chronic obstructive pulmonary disease. Thorax 1999, 54(7):581–586
12. Jones PW, Harding G, Berry P, Wiklund I, Chen W-H, Leidy NK. Development and first validation of the COPD Assessment Test. Eur Respir J. 2009; 34:648–654. DOI: 10.1183/09031936.00102509
13. Vercoulen JHMM, Swanink CMA, Galama JMD, Fennis JFM, van der Meer JWM, Bleijenberg G. Dimensional assessment in chronic fatigue syndrome. J Psychosom Res. 1994; 38:383-392.
14. Vestbo J, Hurd SS, Agusti AG, Jones PW, Vogelmeier C, Anzueto A, et al. Global strategy for the diagnosis, management, and prevention of chronic obstructive pulmonary disease: GOLD executive summary. Am J Respir Crit Care Med. 2013; 187(4):347–365. DOI: 10.1164/rccm.201204-0596PP
15. Worm-Smeitink M, Gielissen M, Bloot L, van Laarhoven HWM, van Engelen BGM, van Riel P, et al. The assessment of fatigue: Psychometric qualities and norms for the Checklist individual strength. J Psychosom Res. 2017; 98:40-46. DOI: 10.1016/j.jpsychores.2017.05.007
16. Hassan M, Mourad S, Wahab NHA, Daabis R, Younis G. Effect of comorbidities on response to pulmonary rehabilitation in patients with chronic obstructive pulmonary disease. The Egyptian Society of Chest Diseases and Tuberculosis. 2016; 65:63-69. <http://dx.doi.org/10.1016/j.ejcdt.2015.11.006>
17. Kon SSC, Canavan JL, Jones SE, Nolan CM, Clark AL, Dickson MJ, et al. Minimum clinically important diﬀerence for the COPD Assessment Test: a prospective analysis. Lancet Respir Med. 2014; 2:195–203. <http://dx.doi.org/10.1016/S2213-2600(14)70001-3>
18. Rebelo P, Oliveira A, Andrade L, Valente C, Marques A. Minimal clinically important differences for patient-reported outcome measures of fatigue in patients with COPD after pulmonary rehabilitation. Chest. 2020; 158(2):550-561. <https://doi.org/10.1016/j.chest.2020.02.045>
19. Yang LL, Yang T. Pulmonary Rehabilitation for Patients with Coronavirus Disease 2019 (COVID-19). Chronic Dis Transl Med. 2020 May 14;6(2):79–86. DOI: 10.1016/j.cdtm.2020.05.002.
20. Leochico CFD. Adoption of tele-rehabilitation in a developing country before and during the COVID-19 pandemic. Ann Phys Rehabil Med. 2020. DOI: 10.1016/j.rehab.2020.06.001
21. Spruit MA, Holland AE, Singh SJ, et al. COVID-19: Interim Guidance on Rehabilitation in the Hospital and Post-Hospital Phase from a European Respiratory Society and American Thoracic Society-coordinated International Task Force. Eur Respir J 2020; in press (<https://doi.org/10.1183/13993003.02197-2020>).
22. British Thoracic Society. Delivering rehabilitation to patients surviving COVID-19 using an adapted pulmonary rehabilitation approach – BTS guidance [Internet]. 16 September 2020, V1.1 [accessed 7 November 2020]. Available from: <https://www.brit-thoracic.org.uk/document-library/quality-improvement/covid-19/pulmonary-rehabilitation-for-covid-19-patients/>
23. Rosen, K., Patel, M., Lawrence, C. et al. Delivering Telerehabilitation to COVID-19 Inpatients: A Retrospective Chart Review Suggests It Is a Viable Option. HSS Jrnl (2020). <https://doi.org/10.1007/s11420-020-09774-4>
24. Spruit MA, Singh SJ, Garvey C, ZuWallack R, Nici L, Rochester C, et al. An official American Thoracic Society/European Respiratory Society statement: key concepts and advances in pulmonary rehabilitation. Am J Respir Crit Care Med. 2013; 188(8): e13–64. DOI: 10.1164/rccm.201309-1634ST
25. British Thoracic Society. Pulmonary Rehabilitation Remote Assessment – version 1 [Internet]. 2020, Apr 22 [Cited 2020, Aug 8]. Available from: <https://www.brit-thoracic.org.uk/document-library/quality-improvement/covid-19/bts-pulmonary-rehab-remote-assessment/>
